# Intraspecific trait variation and adaptability of *Stipa krylovii*: Insight from a common garden experiment with two soil moisture treatments

**DOI:** 10.1002/ece3.10457

**Published:** 2023-08-31

**Authors:** Yulin Liu, Baijie Fan, Ziqing Gong, Luoyang He, Lei Chen, Anzhi Ren, Nianxi Zhao, Yubao Gao

**Affiliations:** ^1^ Department of Plant Biology and Ecology, College of Life Science Nankai University Tianjin China

**Keywords:** adaptation, intraspecific trait variation, Inner Mongolia, plant trait networks, *Stipa krylovii*, trait‐climate relationship

## Abstract

Understanding patterns of intraspecific trait variation can help us understand plant adaptability to environmental changes. To explore the underlying adaptation mechanisms of zonal plant species, we selected seven populations of *Stipa krylovii*, a dominant species in the Inner Mongolia Steppe of China, and evaluated the effects of phenotypic plasticity and genetic differentiation, the effects of climate variables on population trait differentiation, and traits coordinated patterns under each soil moisture treatment. We selected seeds from seven populations of *S. krylovii* in the Inner Mongolia Steppe, China, and carried out a soil moisture (2) × population origin (7) common garden experiment at Tianjin City, China, and measured ten plant traits of *S. krylovii*. General linear analyses were used to analyze how soil moisture and population origin affected each trait variation, Mantel tests were used to analyze population trait differentiation—geographic distance (or climatic difference) relationships, regression analyses were used to evaluate trait‐climatic variable relationships, and plant trait networks (PTNs) were used to evaluate traits coordinated patterns. Both soil moisture and population origin showed significant effects on most of traits. Aboveground biomass, root‐shoot ratio, leaf width, specific leaf area, and leaf nitrogen (N) content were significantly correlated with climate variables under the control condition. Specific leaf area and leaf N content were significantly correlated with climate variables under the drought condition. By PTNs, the hub trait(s) was plant height under the control condition and were aboveground biomass, root length, and specific leaf area under the drought condition. This study indicates that both phenotypic plasticity and genetic differentiation can significantly affect the adaptability of *S. krylovii*. In addition, soil moisture treatments show significant effects on trait‐climate relationships and traits coordinated patterns. These findings provide new insights into the adaptive mechanisms of zonal species in the semiarid grassland region.

## Graphical abstract

This article provides that both quick environmental changes (soil moisture) and long evolutionary history (population origin) can significantly affect adaptability of *Stipa krylovii*, and that the environmental changes can also affect traits coordinated patterns. These findings provide new insights to the adaptive mechanisms of distribution change of dominant species of zonal vegetation.

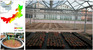

## 1 INTRODUCTION

The adaptability of a plant species along environmental gradient plays an important role in affecting its evolutionary processes and geographical distribution (Ren et al., 2020; Siefert et al., 2015). In the past decades, plant traits have been proven powerful to assess the adaptability of a species to the changes in environment (Wright et al., 2004). From the long‐term point of view, plants can mold their traits to a certain environmental condition by genetic differentiation (natural selection), which could reflect the evolutionary adaptation history of a species (Violle et al., 2007). As a result, plants usually show significant intraspecific trait variation among populations for a certain species along environmental gradients (i.e., light, water, and nutrients) (Siefert et al., 2015). However, plants are susceptible to changing environmental conditions because of their sessile lifestyle (Anderson et al., 2011). From the short‐term point of view, most plants can adjust adaptive strategies (resource acquisition and investment) quickly by phenotypic plasticity and show significant trait differences facing different environmental conditions (Wellstein et al., 2013). Moreover, trait responses to changes in environmental conditions are species‐specific, population‐specific, and trait‐specific (Roybal & Butterfield, 2019; Wang et al., 2015). For example, populations from moist regions show high plasticity in water use efficiency traits; while those in arid regions may show high plasticity in drought‐tolerance traits (Ahrens et al., 2020; Heschel et al., 2004). Therefore, to better predict a species' evolutionary and distribution trends in the context of current climate changes, it is critical for us to understand its phenotypic plasticity (how the quick environmental changes affect the performance of traits) and whether these changes were influenced by their local habitat conditions.

Geographical distances (barriers) (Malécot, 1975) and climatic differences (Schwaegerle & Bazzaz, 1987) are regarded as main factors driving population differentiation. Across the distribution region of a species, geographical distances (barriers) cause population differentiation through limited gene flow between populations. Researchers have found significant correlations between population trait differentiation and geographical distances in natural habitats or common gardens (Adamo et al., 2020; Baruch et al., 2018). On the contrary, due to habitat fragmentation and rapid climate change, the contributions of climatic difference to population trait differentiation are lower than expected when considering multiple traits simultaneously (Jump & Peñuelas, 2005; Wright et al., 2005). However, significant regression relationships between a single trait (such as leaf dry matter content, specific leaf area or leaf N content) and a single local climate variable (such as mean annual temperature, annual precipitation or precipitation variability) were proposed and found useful to explain the adaptability of plant species (Vila‐Cabrera et al., 2015; Wright et al., 2004, 2005).

With the development of a trait‐based framework in the field of ecology, exploring relationships among multiple traits to understand plant adaptation and responses to environmental changes have attracted great interests from ecologists due to the multiple functions and intercorrelations of plant traits (He et al., 2020; Kleyer et al., 2019). Plant traits networks (PTNs) analysis provides a multidimensional approach for describing the overall relationship between multiple traits and identifying the most important trait by quantifying node parameters (He et al., 2020). PTNs analysis has been widely used to study the adaptability among different plant growth forms or among different life forms (Kleyer et al., 2019; Li et al., 2021) and has been gradually used to study the plant population adaptation strategies to rapid environmental changes in recent years (Zhang et al., 2023).

Population responses to environmental changes showing differences are common in zonal vegetation (Duan et al., 2021). Zonal vegetation refers to vegetation with zonal distribution on the earth's surface, which is adapted to local water and heat conditions and can fully reflect the climatic characteristics of a regional vegetation type (Lashchinskiy, 2021). In the semiarid steppe of Inner Mongolia, China, the zonal vegetation communities are meadow steppe, typical steppe, and desert steppe from the east (high precipitation) to the west (low precipitation). *Stipa krylovii* is one of the dominant species in typical steppe, desert steppe, and the gentle slopes of a plateau of meadow steppe. Due to the increasing aridity in the semiarid steppe region in the past decades, the distribution region of *S. krylovii* has gradually expanded eastward because of its relatively stronger drought‐tolerance (Chen et al., 2013; Liu, 2004). Previous studies have shown that both genetic differentiation and phenotypic plasticity dominate intraspecific trait variation of *S. krylovii* either in natural habitats or in a common garden experiment (Zhao et al., 2006, 2016). However, few studies have explored how traits responses of *S. krylovii* populations to aridity (or the drought‐tolerant functional strategy of *S. krylovii*) affected the relationships between traits and climatic variables or traits coordinated patterns. Therefore, exploring how traits of *S. krylovii* are affected by aridity (soil moisture) and whether the population origins affect the effects of aridity on traits would advance our understanding of its adaptive strategies and distribution region changes. Besides, exploring how soil moisture affects the correlations among traits or those between climatic variables and traits of *S. krylovii* is helpful for us to understand and predict this species evolutionary potential in the context of increasing aridity.

In this study, we selected seeds of seven *S. krylovii* populations along a precipitation gradient in its distribution region as study materials and carried out a soil moisture (2) × population origin (7) common garden experiment. Ten traits of *S. krylovii* and their plasticity indices across two soil moisture treatments were measured. We explored how these traits were affected by soil moisture, population origin, and their interaction, and further analyzed how soil moisture affected the correlations among traits and the relationships between climatic variables and traits. Specifically, we proposed the following hypotheses based on the existing knowledge. Firstly, both genetic differentiation caused by population origin and phenotypic plasticity caused by soil moisture treatment would affect the population trait differentiation of *S. krylovii*, but their relative contributions would show trait‐specific (Roybal & Butterfield, 2019). Secondly, population trait differentiation of *S. krylovii* would not be related to geographic distance or climatic difference, while significant regression relationships between population traits (leaf dry matter content, specific leaf area, leaf N content, etc.) and their local climatic variables would be common (Liu et al., 2010; Wang et al., 2015; Wright et al., 2004). Thirdly, traits coordinated patterns of *S. krylovii* would be significantly affected by soil moisture treatment, with drought‐tolerance strategy determining the important node characteristics and overall characteristics under the drought condition.

## 2 MATERIALS AND METHODS

### 2.1 Study species and region

*Stipa krylovii* is a C_3_ bunchgrass, mainly distributed in the typical steppe area of Inner Mongolia, extending to a small part of the meadow steppe in the east, and colonizing part of the desert steppe in the west (Liu, 2004). The mature individual of *S. krylovii* has very dense tussocks about 20 cm in height with long, thin leaves and very dense thin roots concentrated in the 0–20 cm soil layer. It is wind‐pollinated, flowers in mid to late July, ripens in late August or early September, and withered in mid‐October.

**FIGURE 1:**
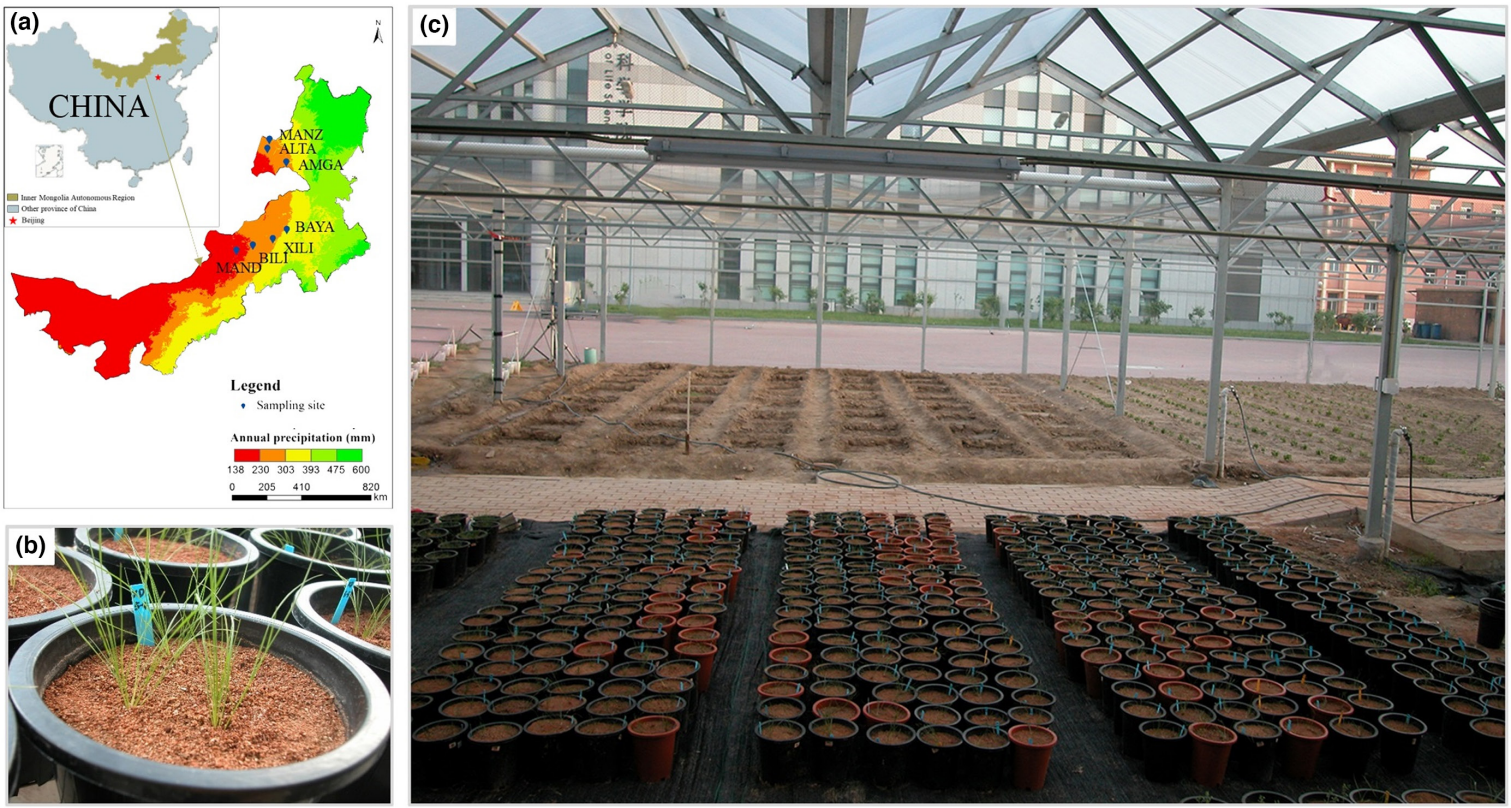
Sample sites of seven *Stipa krylovii* populations (a), seedling morphology (b) and seedling growth environment of common garden experiment (c).

In *S. krylovii* main distribution region, seven representative sites along the precipitation gradient were selected (Figure 1; Table 1), seeds of *S. krylovii* were collected from these sites and used for population construction in the subsequent experiment. Among them, the BAYA population is from the edge of the meadow steppe with a relatively humid environment; MANZ, ALTA, AMGA, and XILI populations are from the typical steppe with moderate precipitation conditions; and BILI and MAND populations are from the desert steppe with drought environment (Table 1; Figure 1a). Geographically, MANZ, ALTA, and AMGA populations located in the eastern of its distribution region, and the other four populations located in the western and middle of its distribution region (Figure 1a).

**TABLE 1:** Ecogeographic characteristics of *Stipa krylovii* seed sampling sites in this study.

Population origin code	Location	Community characteristics	Annual mean temperature (°C)	Annual precipitation (mm)	Temperature seasonality	Precipitation seasonality	Interannual variation of precipitation (1990–2009)
BAYA	44.63° N, 117.73° E	Edge of meadow steppe	1.11	318.00	1433.52	113.06	0.226
MANZ	49.51° N, 117.26° E	Typical steppe	−0.60	286.00	1559.36	120.99	0.232
ALTA	49.85° N, 116.87° E	Typical steppe	1.05	235.00	1579.22	116.81	0.236
AMGA	48.19° N, 118.37° E	Typical steppe	−0.35	281.00	1639.12	118.46	0.238
XILI	44.00° N, 115.07° E	Typical steppe	1.20	293.00	1447.37	111.77	0.214
BILI	43.84° N, 113.82° E	Desert steppe	1.46	232.00	1510.57	117.70	0.216
MAND	44.63° N, 117.73° E	Desert steppe	2.59	218.00	1469.71	111.94	0.218

Five climatic variables of each seed sampling site, including the annual mean temperature, annual precipitation, temperature seasonality, precipitation seasonality, and interannual variation of precipitation, were selected following previous findings (Zhao et al., 2016), and were used for the correlation analysis with plant traits. The first four variables were downloaded from WorldClim (https://www.worldclim.org/), and the interannual variability of precipitation, that is, the coefficient of variation (CV) of precipitation, was calculated by the ratio of standard deviation to mean of 20 years (from 1990 to 2009).

### 2.2 Common garden experiment

A two‐factor (soil moisture × population origin) common garden experiment was carried out at Nankai University (39.10° N, 117.16° E). The experiment was established in plastic pots (20 cm in diameter and 21 cm in height), with a mixture of vermiculite and nutrient soil (1:1) as culture substrate, soil moisture condition was quantified by volumetric water content (VWC). Considering that the water retention of nutrient soil was weaker than that of the local habitat soil in Inner Mongolia Steppe, we set 25% VWC for control treatment and 8% VWC for drought treatment, supplying an amount of water equal to transpiration losses every other day. The second factor was population origin, including seven different populations (Table 1, Figure 1a). Each treatment included 10 pots, with two 6‐month healthy seedlings per pot and each seedling with similar sizes (about 10 tillers, uniform height (10 cm), and root length (12 cm)) (Figure 1b). In total, there were 140 pots (2 soil moisture treatments × 7 population origin ×10 replications). To exclude precipitation, all pots were randomly placed under a five‐meter‐height rain‐proof shed (Figure 1c). Soil moisture was monitored daily by an ECH_2_O Check (Decagon Devices, Pullman, WA, USA). During the experiment, all individuals survived and the position of the pots was changed every week to avoid position effects. The experiment lasted 50 days and ended on July 10, 2009.

### 2.3 Trait measurement

On the 30th day of the experiment, 10 traits related to resource acquisition and investment were measured following standard protocols, with at least 10 replications per treatment. Plant height per pot was the distance between the highest stem leaf and ground level. Fully expanded healthy leaves were used for the measurement of leaf traits. Leaf length, length width, and leaf area per leaf were measured using a Li‐3100 leaf area meter (Li‐Cor, Inc. Lincoln, NE, USA), and the leaf dry mass was weighed after 72 h in an oven at 60°C. Specific leaf area was determined as leaf area / dry mass. Dried leaves were ground for the measurement of leaf N content using an elemental analyzer (Vario‐MAX C/N‐Macro‐elemental analyzer, Germany). The clean fresh roots per pot were used for the measurement of root length after harvest using the WinRHIZO root analysis system (EPSON A3 Transparency Unit / EU‐88). The ratio of root length to belowground biomass was calculated as specific root length.

At the end of the experiment, the shoots and roots were carefully harvested by pot and dried in an oven at 60°C for 72 h to obtain the aboveground biomass and the belowground biomass. The ratio of belowground biomass to aboveground biomass was calculated as the root‐shoot ratio (RSR).

For any trait from the same population origin, plasticity index (*PI*) was quantified by using the trait values calculated under both soil moisture treatments with the following formula (Valladares et al., 2006). The *PI* ranges from 0 to 1, with 0 for no plasticity and 1 for the highest plasticity.

$$\mathrm{PI}=\frac{\mathrm{Max}\;\left[\text{treatment}\left(\text{control},\text{drought}\;\right)\right]-\mathrm{Min}\;\left[\text{Treatment}\left(\text{control},\text{drought}\right)\right]}{\mathrm{Max}\;\left[\text{Treatment}\left(\text{control},\text{drought}\right)\right]}$$

### 2.4 Statistical analyses

Before statistical analyses, we used Box‐Cox transformation for aboveground biomass, belowground biomass, leaf length, specific leaf area, leaf N content, and specific root length to make the data fit the normal distribution. In addition, the significant difference in trait average values among populations were tested by Fisher's least significant difference (LSD) of multiple comparison.

### 2.5 Effects of soil moisture, population origin, and their interaction on traits

To analyze the effects of soil moisture, population origin, and their interaction on all traits, the general linear models (GLM) were performed using the *lm* function in R (R 4.1.1), with soil moisture and population origin as fixed factors, and initial tillers as a covariable. Variance explained by each factor was calculated by the mean square of each factor into its variance component (Graham & Edwards, 2001).

### 2.6 Correlation between geographical distance/climatic difference matrix and trait differentiation matrix

In order to investigate whether population trait differentiation is affected by the geographical distance or climatic difference of population origin, we calculated the geographical distance matrix and climatic difference matrix among seed sampling sites. The geographical distance (km) was calculated according to the longitude and latitude of sampling sites using the *distm* function of the “*geosphere*” package (version 1.5‐18). The climate difference matrix was calculated by Euclidean distance between populations after *z*‐score standardizing of the five climatic variables (annual average temperature, annual precipitation, interannual precipitation variability, temperature seasonality, and precipitation seasonality) using the *dist* function of the “*geosphere*” package (version 1.5–18). Population trait differentiation was also calculated by Euclidean distance using the *dist* function of the “*geosphere*” package (version 1.5–18) for data collected under each soil moisture treatment.

Finally, the Mantel test was used to test the correlation of trait‐geographic distance matrixes, and trait‐climate difference matrixes using the *Pearson* function of the “*vegan*” package (version 2.6‐4).

### 2.7 Regression relationships between a single climatic variable and a single trait value

The relationship between each trait measured under each soil moisture treatment and each climate variable in Table 1 was analyzed by linear regression analysis. Considering that the relationship between a single trait and a single environmental variable may be linear or nonlinear (Manzaneda et al., 2015), we considered both the first‐order and the second‐order polynomial relationships between them using the *lm* and *polynomial* functions in R, with climate variables as independent variables and traits as dependent variables.

### 2.8 Relationships among traits

Plant trait networks (PTNs) enable us to observe association among plant traits. We used it to analyze traits coordinated patterns by the package “*igraph*” (version 1.4.1) in R. Before the analyses, correlations between traits are tested by Pearson correlation.

For the overall characteristics of PTNs, the edge density (*ED*), diameter (*D*), average path length (*AL*), and average clustering coefficient (*AC*) were selected as the key parameters (He et al., 2020). For the node characteristics of PTNs, the degree (*k*), closeness (*C*), and betweenness (*B*) were selected as the key parameters (He et al., 2020).

$$\mathrm{ED}=\frac{2L}{n∙\left(n-1\right)}$$

$$D=\max\left\{d_{\mathrm{ij}}\right\}$$

$$\mathrm{AL}=\frac{1}{n\left(n-1\right)}\sum i\neq j\;d_{\mathrm{ij}}$$

$$\mathrm{AC}=\frac{1}{n}\sum_{i=1}^{n}{\mathrm{CC}}_{i}$$

$$k_{i}=\sum_{j\neq i}a_{\mathrm{ij}}$$

$$C_{i}=\frac{n-1}{\sum_{j=1}^{n-1}d_{\mathrm{ij}}}\left(i\neq j\right)$$

$$B_{i}=\sum_{\mathrm{jk}}\sigma \left(j,i,k\right)$$

*a*_*ij*_ is the connection between the focal node trait *v*_*i*_ and node trait *v*_*j*_; *L* is the number of actual edges of the network; *n* is the number of node traits; *d*_*ij*_ is the shortest distance between focal node trait *v*_*i*_ and node trait *v*_*j*_; CC_*i*_ is the clustering coefficient of focal node trait *v*_*i*_.

1. The edge density (*ED*) describes the density of edges connecting traits, and a PTN with a higher edge density (*ED*) may have more efficient resource utilization and integration.
2. The diameter (*D*) and the average path length (*AL*) are the maximum value and the average value of the shortest path length between any two traits, respectively. A PTN with a higher *D* or a higher *AL* means higher independence among traits, which is less easy to form functional clustering.
3. The average clustering coefficient (*AC*) is the average of clustering coefficients of all traits, and a PTN with a higher *AC* means more possibility to form traits' clusters to achieve specific functions.
4. The degree (*k*) is the number of connections in which a trait node is significantly related to other traits. A trait with the highest *k* in a PTN can interact with more other traits and further adjust the whole traits network, and is called a hub trait.
5. The closeness (*C*) is the reciprocal of the shortest mean path between a trait and another trait. A trait with a relatively higher *C* indicates that the trait is more closely related to other traits.
6. The betweenness (*B*) of a trait is the number of all the shortest paths through the trait. A trait with the highest *B* in a PTN can play the role of “mediators” in connecting coordinates several subnetworks or functional clusters, and is called mediator trait.

## 3 RESULTS

### 3.1 Plant trait responses to soil moisture and population origin

The trait average values per population and their differences among populations by LSD test were shown in Table S1; in addition, their plasticity indices across treatments were shown in Table S2. The factor of soil moisture (indicating phenotypic plasticity) had significant effects on all traits and showed the greatest contribution to variance in plant height (Table 2). The factor of population origin (indicating genetic differentiation) had significant effects on all traits except for root‐shoot ratio and root length and showed the greatest contribution to variance in leaf width (Table 2). The interaction between soil moisture and population origin had significant effects on aboveground biomass, leaf width, and leaf N content (Table 2).

**TABLE 2:** The effects of soil moisture, population origin and their interaction on each trait and the variance explained by each unique factor.

Traits	Soil moisture (df = 1)			Population origin (df = 6)			Soil moisture × population origin (df = 6)		
	*F*	*p‐*value	Variance explained (%)	*F*	*p‐*value	Variance explained (%)	*F*	*p‐*value	Variance explained (%)
Aboveground biomass	84.38	**<.001**	34.0	3.43	**.004**	5.9	2.25	**.045**	3.1
Belowground biomass	81.41	**<.001**	32.6	5.35	**<.001**	10.6	1.27	.278	0.1
Root‐shoot ratio	9.97	**.002**	5.9	1.47	.197	1.9	0.93	.477	0.0
Plant height	131.14	**<.001**	43.6	5.73	**<.001**	9.5	0.39	.882	0.0
Leaf length	105.22	**<.001**	35.1	8.54	**<.001**	17.8	1.08	.375	0.0
Leaf width	40.58	**<.001**	16.4	9.47	**<.001**	24.6	2.77	**.014**	0.7
Specific leaf area, SLA	13.55	**<.001**	7.4	3.82	**.002**	9.9	1.67	.139	0.4
Leaf N content	29.23	**<.001**	15.1	3.97	**.002**	10.7	2.74	**.018**	0.9
Root length	30.20	**<.001**	5.8	0.80	.571	0.0	1.22	.301	0.0
Specific root length, SRL	49.30	**<.001**	24.3	3.48	**.003**	7.6	0.49	.816	0.0

*Note:* Values in bold style indicate the significant effects (*p*‐value < .05).

### 3.2 Relationships between population trait differentiation and geographic distance/climate difference

No significant correlations were found between the population trait differentiation matrix and their geographic distance / climate difference matrix by Mantel test (Table 3; Tables S3 and S4).

**TABLE 3:** The correlations (*r*) and significance (*p*‐value) between population trait differentiation matrix (calculated by Euclidian distance) and their geographical distance/climatic difference by Mantel test.

Treatment	Geographic distance		Climatic difference	
	*r*	*p*‐value	*r*	*p*‐value
Control	0.21	.158	0.27	.085
Drought	0.21	.105	0.11	.252

**FIGURE 2:**
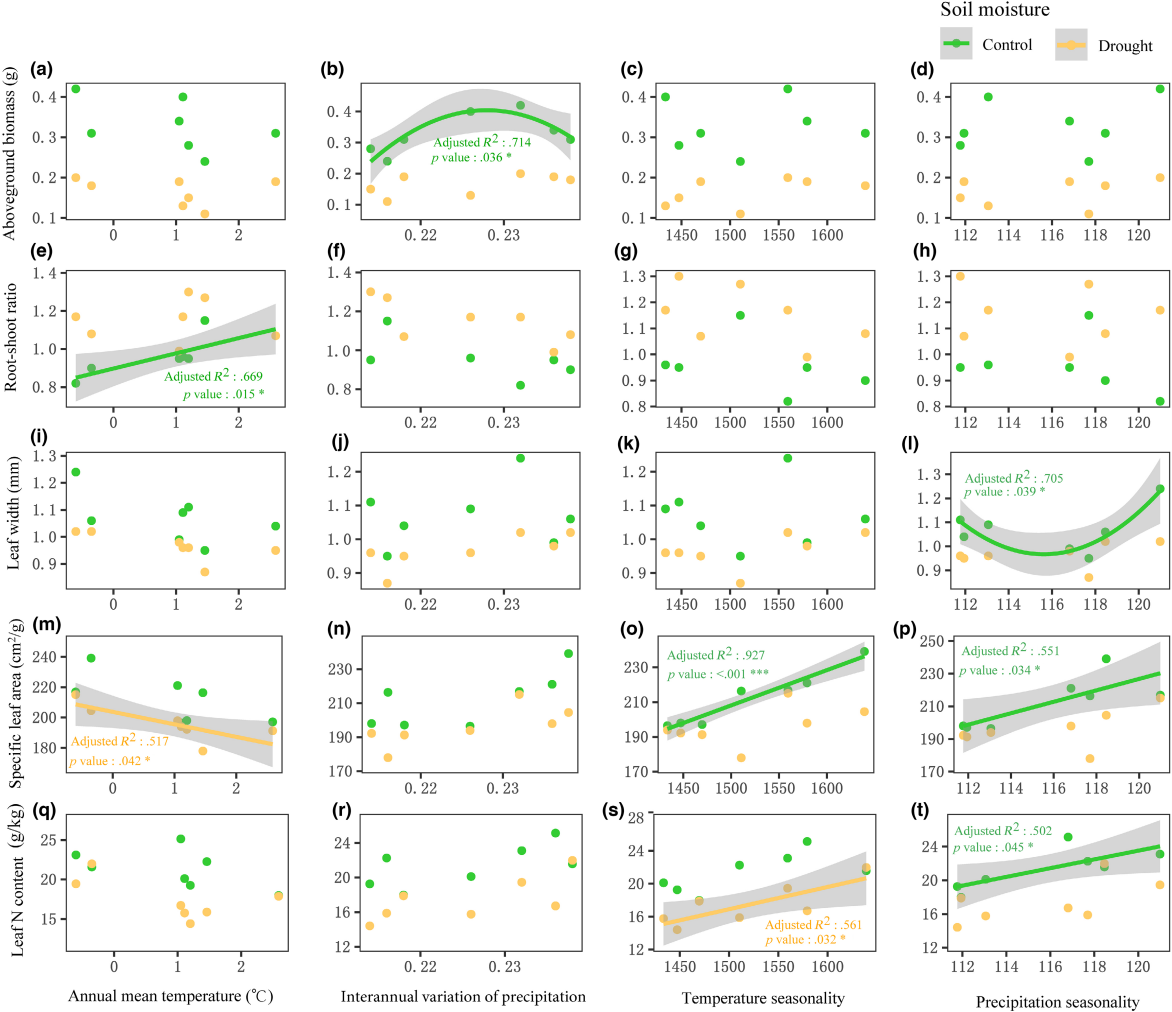
Regression analysis between traits of *Stipa krylovii* observed in the common garden experiment and climatic variables. A regression lines and 95% confidence interval were shown when a regression relationship is significant (adjusted *R*‐squared and *p*‐value). The annual precipitation which was not significantly associated with any trait and belowground biomass, plant height, leaf length, root length, and specific root length which were not significantly related to any climatic variable were not shown in subfigures, and the detail results were shown in Table S5.

Five traits (aboveground biomass, root‐shoot ratio, leaf width, specific leaf area, leaf N content) out of the ten traits revealed significant regression relationships with at least one climatic variable, while no traits showed significant regression relationships with the annual precipitation (Figure 2a–t; Table S5).

Under the control condition, aboveground biomass showed a convex relationship with the interannual variation of precipitation (Figure 2b). Root‐shoot ratio revealed a positive relationship with annual mean temperature (Figure 2e). Leaf width revealed a concave relationship with precipitation seasonality (Figure 2l). Specific leaf area showed positive relationships with temperature seasonality (Figure 2o) and precipitation seasonality (Figure 2p). Leaf N content revealed a positive relationship with precipitation seasonality (Figure 2t).

Under the drought condition, specific leaf area had a significant negative relationship with annual mean temperature (Figure 2m), and leaf N content revealed a significant positive relationship with temperature seasonality (Figure 2s).

### 3.3 Traits coordinated patterns

**FIGURE 3:**
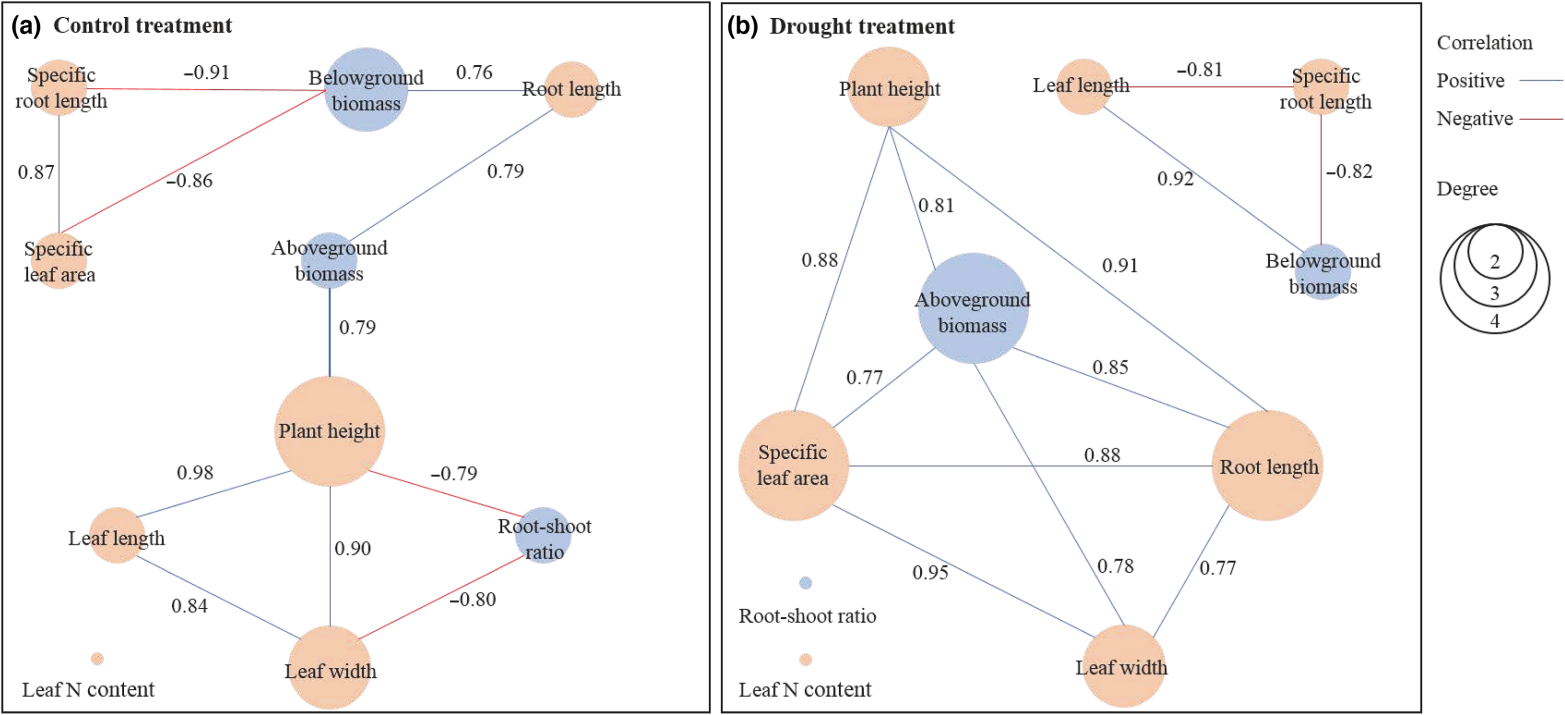
Plant traits networks and traits coordinated patterns obtained under the control condition (a) and the drought condition (b) by PTNs analysis. The blue circles represent biomass traits and the red circles represent functional traits. The numbers on the line represent the correlation coefficients. The size of the circle represents the degree parameter of the trait in PTNs.

The correlation coefficients and significance between any pairwise traits were shown in Table S6, and traits coordinated patterns (Figure 3a,b) by PTNs showed only the significant correlations under each treatment (control and drought). Under the control condition, 11 out of 45 pairwise traits showed significant relationships, with seven relationships being positive and four relationships being negative (Figure 3a). Under the drought condition, 12 correlations showed significant relationships, with 10 relationships being positive and two relationships being negative (Figure 3b), which was significantly different from the findings under the control condition (Figure 3a).

For overall characteristics of PTNs, *ED* and *AC* were lower under the control condition (0.305 and 0.593) than those under the drought condition (0.428 and 0.938); while the *D* and *AL* were higher under the control condition (5 and 2.639) than those under the drought condition (2 and 1.077) (Table 4).

**TABLE 4:** Key parameters of overall characteristics in plant trait networks (PTNs).

Overall parameters	Control	Drought
Edge density (*ED*)	0.305	0.428
Diameter (*D*)	5	2
Average path length (*AL*)	2.639	1.077
Average clustering coefficient (*AC*)	0.593	0.938

For key parameters of node traits in PTNs, hub traits or mediator traits were different between both treatments. Under the control condition, plant height showed the highest degree (*k* = 4), and aboveground biomass showed the highest closeness (*C*_𝑖_ = 0.063) and betweenness (*B*_*i*_ = 16) among all traits. Under the drought condition, aboveground biomass, root length, and specific leaf area showed the highest values for degree, closeness, and betweenness among all traits (Table 5).

**TABLE 5:** Key parameters of node characteristics in plant trait networks (PTNs). (The maximum value(s) within a column is marked by bold).

Traits	Degree (*k*)		Closeness (*C*)		Betweenness (*B*)	
	Control	Drought	Control	Drought	Control	Drought
Aboveground biomass	2	**4**	**0.063**	**0.036**	**16.00**	**0.33**
Belowground biomass	3	2	0.050	0.024	12.00	0.00
Root‐shoot ratio	2	0	0.043	0.000	0.00	0.00
Plant height	**4**	3	0.059	0.034	15.50	0.00
Leaf length	2	2	0.043	0.024	0.00	0.00
Leaf width	3	3	0.045	0.034	0.50	0.00
Specific leaf area	2	**4**	0.038	**0.036**	0.00	**0.33**
Leaf N content	0	0	0.000	0.000	0.00	0.00
Root length	2	**4**	0.059	**0.036**	15.00	**0.33**
Specific root length	2	2	0.038	0.024	0.00	0.00

## 4 DISCUSSION

In a soil moisture × population origin common garden experiment, the results showed that both soil moisture and population origin significantly affected most traits of *S. krylovii* (Table 2), confirming that both quick adaptive strategies (phenotypic plasticity) and evolutionary history (genetic differentiation) jointly affected intraspecific trait variation of *S. krylovii*, supporting the first hypothesis. Significant regression relationships were found between some plant traits and climate variables (Figure 2) and no significant correlations were found between the population trait differentiation matrix and geographic distance/climatic difference matrix by Mantel tests, which is consistent with the second hypothesis (Table 3). The results of PTNs revealed significant differences in traits coordinated patterns between the two soil moisture treatments (Figure 3), supporting the third hypothesis to some extent. These findings provide novel insights for us to understand the distribution trends and evolutionary potential of *S. krylovii*.

### 4.1 Phenotypic plasticity and genetic differentiation

The finding that soil moisture explained more variance than population origin in eight out of 10 traits indicated that phenotypic plasticity rather than genetic differentiation played a more important role in affecting intraspecific trait variation in *S. krylovii* (Table 2). By a model study, Edelaar et al. (2017) have mentioned that genetic differentiation is the default adaptation mechanism in small environmental fluctuations while phenotypic plasticity rather than genetic differentiation plays a more important role in determining species adaptability in a severe environmental fluctuation. Based on this opinion, the findings in this study could explain the eastward shift of *S. krylovii* distribution region in recent decades in the context of increasing aridity. Some other studies have also indicated that phenotypic plasticity plays a dominant role in affecting adaptation mechanisms of some herbaceous plants (Gonzalo‐Turpin & Hazard, 2009; Lajoie & Vellend, 2018). Furthermore, the plasticity indices of the biomass, specific root length, biomass allocation, plant height, and leaf length were relatively higher than those of the others (Table S2), suggesting that these traits were important for the adaptability of *S. krylovii* that colonizes in patchy environments with strong heterogeneity (Matesanz et al., 2010).

The finding that genetic differentiation (population origin effect) showed the highest contribution to variance in leaf width and specific leaf area (Table 2) demonstrated that leaf morphology and structure were more likely to be affected by genetic differentiation. Similar results have been found in previous studies (Gonzalo‐Turpin & Hazard, 2009; Hovenden & Schoor, 2003; Lajoie & Vellend, 2018). All these findings were consistent with the theory of the “global leaf economic spectrum” that has revealed population genetic differentiation in leaf traits (Reich, 2014; Wright et al., 2004).

### 4.2 Relationships between climate variables and population trait

Under the control condition, the finding that populations from higher temperature seasonality and precipitation seasonality had higher specific leaf area and higher leaf N content indicated that these populations are related to faster acquisition of light and nutrient resources. In recent years, many studies have shown the important role of seasonal climate in driving plant trait differentiation, especially specific leaf area of grasses (Grant et al., 2017; Keep et al., 2021; Williams et al., 2017). Variations in precipitation and dry season length may cause plant leaf trait differentiation by different selective pressures (Moore et al., 2020). In regions with high seasonal drought, plants with high specific leaf area would take advantage of temporary favorable conditions through rapid growth and carbon accumulation (Kikuzawa et al., 2013).

The finding that plant traits as well as their regression relationships with climate variables were significant different between two soil moisture treatments was consistent with previous findings (Eziz et al., 2017; Rodríguez‐Alarcón et al., 2022). Specific leaf area was significantly related with annual mean temperature under the drought condition (Figure 2m) but with temperature and precipitation seasonality under the control condition, which implied that local annual mean temperature shaped specific leaf area strongly under relatively arid conditions. Similarly, Yu et al. (2022) have revealed that specific leaf area tends to decline when plants are exposed to a combination of rising heat and water deficit. In addition, leaf N content showed a significant correlation with temperature seasonality under the drought condition (Figure 2s) but with precipitation seasonality under the control condition, suggesting that populations from higher temperature seasonality regions were related to higher leaf N content under the drought condition, which is consistent with the role of carbohydrate reserve in cold resistance of perennial grass species (Chatterton et al., 1989).

The finding that root‐shoot ratio was positively correlated with annual mean temperature under the control condition (Figure 2e), which indicated that populations in warmer regions might invest more in the belowground than the aboveground part under the nondrought condition. Domisch et al. (2001) have found that the increase in soil temperature would increase the root‐shoot ratio of *Pinus sylvestris* by promoting the growth of belowground part in a temperature control treatment. Kummerow and Ellis (1984) have shown that photosynthates of *Carex bigelowii* are more likely to be allocated to leaf production under lower temperature conditions. Therefore, the findings in this study implied that root systems of *S. krylovii* developed well in relatively higher soil moisture content and warmer regions, which was conducive to new colonization and could provide an explanation for the eastward shift of its distribution region.

The quadratic relationship between aboveground biomass and interannual precipitation variability indicated that the aboveground biomass of *S. krylovii* was limited by precipitation variability and that the highest aboveground biomass was found in regions with moderate interannual variability. In a global synthesis by Gherardi and Sala (2019), the researchers have revealed that from high precipitation region to low precipitation region in the arid land, the effect of interannual precipitation variability to plant aboveground biomass changed from positive to negative. These results indicated that precipitation variability might be the key factor affecting plant aboveground biomass performance in arid and semiarid regions, supporting that precipitation variability, whether too high or too low, was not conducive to plant investment in aboveground biomass (Cheng et al., 2021; Gherardi & Sala, 2019).

### 4.3 Traits coordinated patterns

There were some differences in traits coordinated patterns between control and drought treatments (Figure 3). Under the drought condition, the relatively lower *D* and *AL*, and higher *ED* and *AC* demonstrated that the weaker independence and more efficient resource integration of traits, which suggested that there was more possibility to form functional clustering under the drought condition (Table 4). For example, there were two separate clusters under the drought condition while only one cluster under the control condition (Figure 3). Furthermore, Rodríguez‐Alarcón et al. (2022) have observed that trait filtering effect and functional space clustering are common under the drought condition after studying on 52 herbaceous species under both control and drought treatments. Similarly, in a study on 10 traits' probabilistic graphical models, Flores‐Moreno et al. (2019) have revealed that plants would face more selection and thus show specific ecological functions and trade‐off relationships in a relatively resource‐poor environment.

For specific traits, under the control condition, the plant height which is related to competing for light was the hub trait (Table 5), suggesting that plant height could interact with more other traits; and the aboveground biomass was the mediator trait (Table 5), demonstrating the coordinating role of aboveground biomass among several subnetworks. While under the drought condition, aboveground biomass, specific leaf area, and root length were the hub traits and mediator traits (Table 5), suggesting that plant aboveground growth, leaf light use efficiency and root growth were more closely related. Such changes of traits coordinated patterns were related to different plant response strategies under different soil moisture treatments (de Vries et al., 2016). Under drought stress conditions, plants with higher light capture capability (such as higher SLA) and stronger ability to elongate roots (such as higher root length) can maintain growth, which links to resource acquisition strategies of the whole‐plant economic spectrum (Poorter et al., 2014); while plants with lower specific leaf area and shorter root length can adopt resource conservation strategies to improve drought‐tolerance (Medeiros et al., 2017; Poorter et al., 2014). Thus, the positive relationship between specific leaf area and root length (Figure 3b) could provide an empirical support for the synergy between the leaf and root of the whole plant under the drought stress condition.

## 5 CONCLUSION

Our findings reveal the strong selection effect of seasonal climate on plant leaf traits and the significant differences in traits coordinated patterns between both soil moisture treatments. This study provides new insights to explain the adaptive mechanisms of zonal vegetation in the context of global climate changes and highlights the necessity to account for intraspecific trait variation in the study of species diversity and distribution and in implication for ecological assessment.

## AUTHOR CONTRIBUTIONS

**Yulin Liu:** Conceptualization (equal); data curation (equal); formal analysis (equal); methodology (equal); resources (equal); software (lead); validation (lead); visualization (lead); writing – original draft (lead); writing – review and editing (equal). **Baijie Fan:** Data curation (equal); formal analysis (equal); methodology (equal); resources (equal); visualization (equal); writing – review and editing (equal). **Ziqing Gong:** Data curation (equal); formal analysis (equal); software (equal); validation (equal); writing – review and editing (equal). **Luoyang He:** Data curation (equal); formal analysis (equal); software (equal); visualization (equal); writing – review and editing (equal). **Lei Chen:** Methodology (equal); resources (equal); supervision (equal). **Anzhi Ren:** Conceptualization (equal); data curation (equal); methodology (equal); resources (equal); supervision (equal); writing – review and editing (equal). **Nianxi Zhao:** Conceptualization (equal); data curation (equal); formal analysis (equal); funding acquisition (lead); methodology (equal); project administration (equal); resources (lead); validation (lead); writing – review and editing (equal). **Yubao Gao:** Conceptualization (equal); data curation (equal); methodology (equal); resources (equal); validation (equal).

## FUNDING INFORMATION

This work was supported by the National Natural Science Foundation of China (32171522, 31770505).

## CONFLICT OF INTEREST STATEMENT

All authors had no conflict of interest to declare.

## Supporting information


Appendix S1

## Data Availability

The data that support the findings of this study will be available in Dryad at https://doi.org/10.5061/dryad.69p8cz96z.
